# Dietary Fat Intake and the Risk of Depression: The SUN Project

**DOI:** 10.1371/journal.pone.0016268

**Published:** 2011-01-26

**Authors:** Almudena Sánchez-Villegas, Lisa Verberne, Jokin De Irala, Miguel Ruíz-Canela, Estefanía Toledo, Lluis Serra-Majem, Miguel Angel Martínez-González

**Affiliations:** 1 Department of Clinical Sciences, University of Las Palmas de Gran Canaria, Las Palmas de Gran Canaria, Spain; 2 Department of Preventive Medicine and Public Health, University of Navarra, Pamplona, Spain; 3 Division of Human Nutrition, Wageningen University, Wageningen, The Netherlands; University College Dublin, Ireland

## Abstract

Emerging evidence relates some nutritional factors to depression risk. However, there is a scarcity of longitudinal assessments on this relationship.

**Objective:**

To evaluate the association between fatty acid intake or the use of culinary fats and depression incidence in a Mediterranean population.

**Material and Methods:**

Prospective cohort study (1999–2010) of 12,059 Spanish university graduates (mean age: 37.5 years) initially free of depression with permanently open enrolment. At baseline, a 136-item validated food frequency questionnaire was used to estimate the intake of fatty acids (saturated fatty acids (SFA), polyunsaturated fatty acids (PUFA), *trans* unsaturated fatty acids (TFA) and monounsaturated fatty acids (MUFA) and culinary fats (olive oil, seed oils, butter and margarine) During follow-up participants were classified as incident cases of depression if they reported a new clinical diagnosis of depression by a physician and/or initiated the use of antidepressant drugs. Cox regression models were used to calculate Hazard Ratios (HR) of incident depression and their 95% confidence intervals (CI) for successive quintiles of fats.

**Results:**

During follow-up (median: 6.1 years), 657 new cases of depression were identified. Multivariable-adjusted HR (95% CI) for depression incidence across successive quintiles of TFA intake were: 1 (ref), 1.08 (0.82–1.43), 1.17 (0.88–1.53), 1.28 (0.97–1.68), 1.42 (1.09–1.84) with a significant dose-response relationship (p for trend = 0.003). Results did not substantially change after adjusting for potential lifestyle or dietary confounders, including adherence to a Mediterranean Dietary Pattern. On the other hand, an inverse and significant dose-response relationship was obtained for MUFA (p for trend = 0.05) and PUFA (p for trend = 0.03) intake.

**Conclusions:**

A detrimental relationship was found between TFA intake and depression risk, whereas weak inverse associations were found for MUFA, PUFA and olive oil. These findings suggest that cardiovascular disease and depression may share some common nutritional determinants related to subtypes of fat intake.

## Introduction

Depression affects about 151 million people worldwide [1]. In 2004, unipolar depression was the third leading cause of Disability Adjusted Life Years (DALY's) in the world and the main cause in middle and high income countries [1].

Wide variations exist in the prevalence of depression across countries, suggesting that disparities in the distribution of several risk factors may determine this heterogeneity [2]. Between-country differences in food habits could account for these differences. Epidemiological evidence is accruing in recent years to support a relationship between improved nutrition and better mental health [3]–[7]. Many biological mechanisms give support also to this relationship [8]–[11]. Surprisingly, in spite of the high prevalence of mental disorders, little etiological longitudinal research has been conducted to assess the relationship between nutrition and depressive disorders [3], [4], [12].

Rising secular trends in the incidence of depressive disorders have been paralleled by a dramatic change in the sources of fat in the Western diet. This change mainly consists in the replacement of polyunsaturated (PUFA) or monounsaturated fatty acids (MUFA) by saturated fats (SFA) and trans-unsaturated fats (TFA) [11]. In consistency with this trend, the so-called “Western” food pattern (rich in SFA and TFA and common in Northern Europe and USA) has been reported as a relevant risk factor for depression [13], [14].

Suicide rates and lifetime prevalence of mental disorders in Europe show higher levels in Northern countries and the lowest levels in Southern Mediterranean countries [15], [16]. Although recent trends point towards a progressive narrowing of between-countries differences, food choices of Northern and Southern European countries differ mainly in the consumption of two food items: olive oil and pulses, with very high consumption in Southern Europe [17]. In a previous report by our group, a higher adherence to the Mediterranean food pattern (rich in legumes, fruits, vegetables, fish and cereals, but low in meat and dairy products, and followed in Southern Europe) was found to be inversely associated with the risk of depression [18].

However, beyond adherence to an overall dietary pattern, little is known about the effect of specific types of major fat sources on depression risk. Usually, PUFA [19] and olive oil (OO) [20]–[22] have been considered as healthy lipids because they reduce the incidence of cardiovascular disease (CVD). Contrarily, SFA and, specially, TFA are known risk factors for CVD [23]. There are some reasons to expect that CVD and depression might share some common determinants [24]. However, excepting for fish oils or omega-3 (n-3) fatty acids, the influence of other fat subtypes on depression risk has received only little attention. In trials using fish oils for the treatment of depression, mood scores improved significantly for both the fish oil group and the control group who received OO [25]. A possible explanation was that both fish and OO, or simply an increased fat intake, were equally effective against depressive symptoms, because low fat diets may adversely affect mood [9], [26].

The aim of our study was to assess the role of dietary fat and fat subtypes on depression occurrence in a Mediterranean prospective cohort. To our knowledge, only a recent case-control study [27] and two previous smaller cohorts [28], [29] have analyzed the association between fat subtypes and depression risk. Both previous cohorts reported inverse associations with OO or oleic acid.

## Materials and Methods

### Subjects

**Figure 1:**
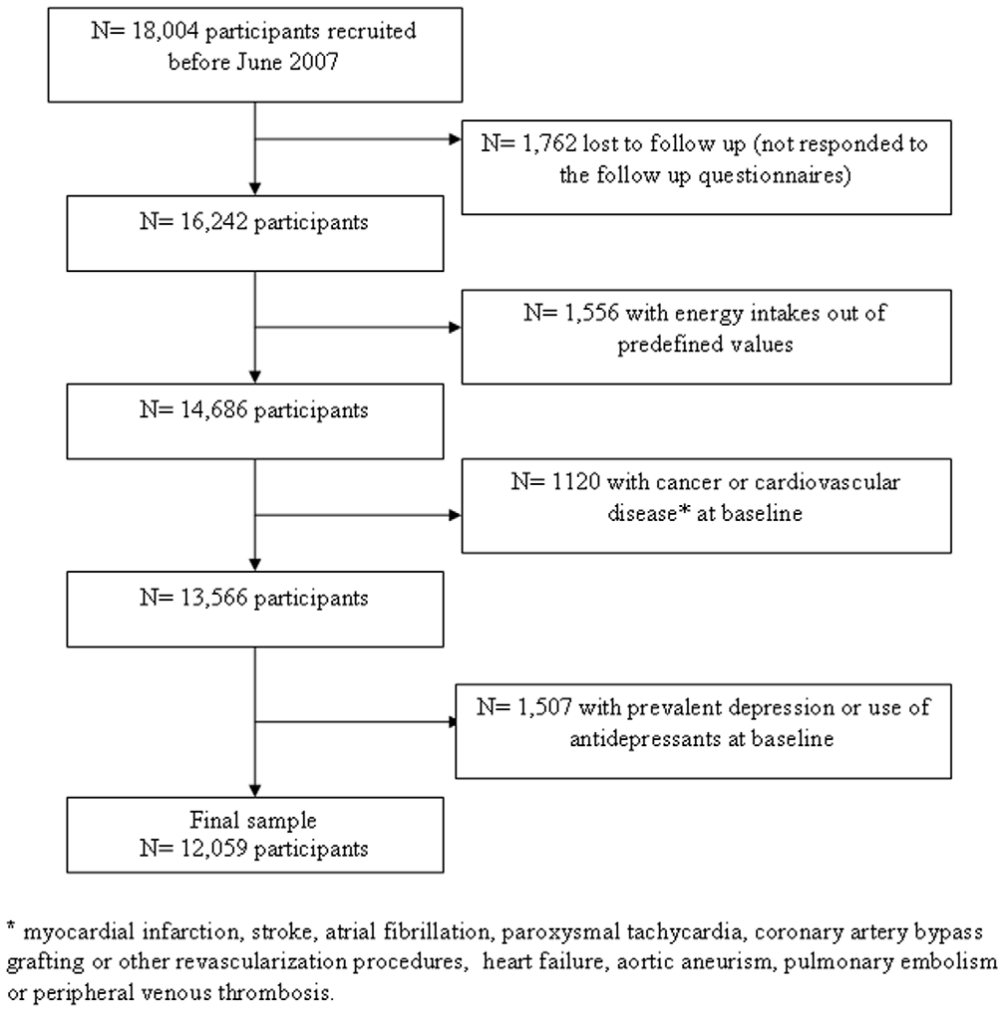
Flow chart of participants. The SUN Project.

The “Seguimiento Universidad de Navarra” (SUN) Project is a dynamic cohort study including only university graduates initiated in December 1999 in Spain and patterned after the models of the Nurses Health Study and the Health Professionals Follow-up Study [23]. Its methods have been previously described elsewhere [30], [31]. Information is gathered biennially by mailed questionnaires. Up to August 2007, 18,004 subjects had completed the baseline questionnaire. Subjects lost to follow up (n = 1,762), with energy intake out of predefined limits (<800 Kcal/day or >4,000 Kcal/day in men and <500 Kcal/day or >3,500 Kcal/day in women) (n = 1,556), with a history of cancer or cardiovascular disease at baseline (n = 1,120), and users of antidepressant medication or with depression at baseline (n = 1,507), were excluded. Finally 12,059 participants who had at least completed one follow-up questionnaire up to May 2010 were included in the analyses (Figure 1).

The study was approved by the Institutional Review Board of the University of Navarra. Written informed consent was not requested to the participants. Voluntary completion of the first questionnaire was considered to imply informed consent. Our Institutional Review Board specifically approved this consent process.

### Exposure assessment

Dietary intake at baseline was assessed through a validated semi-quantitative food-frequency questionnaire [32], [33]. It showed reasonably good validity for assessing fat intake (energy-adjusted intraclass correlation coefficients for different types of fats versus four 3-day food records ranged from 0.49 to 0.75) [33]. Nutrient intakes were calculated using the latest available information included in food composition tables for Spain. Adjustments of nutrients were made for total energy intake by using the residuals method [34]. The ratio n-3/n-6 was also computed [35].

### Outcome assessment

A participant was defined as an incident case of depression if he/she responded positively in a follow-up questionnaire to the question: ‘Have you ever been diagnosed of depression by a medical doctor?’ or who reported the habitual use of antidepressant drugs in at least one of the follow-up questionnaires completed after 2, 4, 6, 8 or 10 years. Antidepressant use was ascertained though an open question in which the participants reported their medication utilization.

A self-reported diagnosis of depression done by a physician has been validated in a sub-sample of our cohort using the Structured Clinical Interview for DSM-IV (SCID-I) as Gold Standard. The estimated sensitivity and specificity were 0.37 and 0.96 respectively [36].

### Covariate assessment

Information on medical, socio-demographic, anthropometric, and life-style related variables were obtained from the baseline questionnaire. Physical activity was assessed through a validated physical activity questionnaire [37]. Adherence to the Mediterranean Dietary Pattern (MDP) was assessed combining 8 items (fruits and nuts, vegetables, fish, legumes, cereals, meat and meat products, dairy and alcohol intake) [38]. To avoid overlapping with our main exposure, we excluded the ratio MUFA/SFA item from this score (the actual range in our study was from 0 to 8).

### Statistics

Cox regression models were used to assess the relationship between quintiles of total dietary fatty acids and culinary fats and the incidence of depression using always the lowest quintile as reference. Tests of linear trend across increasing quintiles were conducted by assigning the medians to each quintile; this variable was treated as continuous. The Cox model used age as the underlying time variable. Birth date was used as the origin variable, entry time was defined as age at recruitment. Exit time was defined as age at diagnosis of depression for cases or age when completing the last follow-up questionnaire or age at death (whichever occurred first) for participants who did not develop depression. We stratified the analyses by age (in 5-year intervals) to control for calendar-period and birth cohort effects [39]. Other potential confounders included in the multiple-adjusted model were: sex, age, smoking, physical activity during leisure time, total energy intake, and baseline body mass index (BMI). In separate models, additional adjustments were made for adherence to the MDP. We conducted sensitivity analyses, and repeated all the analyses 1) after excluding early cases (in the two first years), 2) after excluding late cases (after >8 years), 3) after excluding participants reported taking antidepressant drugs but not a physician diagnosis of depression.

## Results

We assessed 5,038 men and 7,021 women. Mean age at recruitment was 37.5 years (SD: 11.5). The main characteristics of the study population according to extreme quintiles of dietary fat intake are shown in Table 1.

**Table 1:** Baseline characteristics according to extreme quintiles of olive oil consumption and specific types of fat intake, mean (SD).

Characteristic	OO		PUFA		TFA	
	Q1	Q5	Q1	Q5	Q1	Q5
Sex (% men)	59.9	26.0	40.7	43.7	40.1	45.5
Age at baseline (y)	37 (12)	38 (11)	38 (12)	36 (11)	40 (12)	35 (11)
BMI (kg/m2)	24 (3)	23 (4)	23 (3)	23 (4)	24 (4)	23 (3)
Smoking:						
Ex smoker (%)	26.6	35.0	30.4	25.1	35.0	23.4
Current (%)	21.6	23.4	20.6	24.9	19.5	24.0
Marital status (% married)	46.4	54.9	54.0	46.9	54.4	43.8
Unemployed (%)	4.3	5.1	3.9	5.3	3.9	4.4
Leisure time physical activity (Mets-h/w)	28 (26)	23 (20)	27 (26)	23 (21)	27 (25)	24 (22)
Total energy intake (Kcal/d)	2686 (595)	2267 (509)	2616 (534)	2517 (615)	2607 (565)	2495 (574)
Mediterranean Dietary Pattern (0–8)	3.6 (1.6)	4.1 (1.6)	4.3 (1.5)	3.4 (1.6)	4.9 (1.4)	2.9 (1.5)
Total fat intake*	34.7 (6.3)	40.7 (6.8)	31.6 (6.0)	40.8 (5.8)	32.8 (6.9)	40.2 (5.4)
Saturated fat intake*	13.0 (3.3)	12.1 (3.0)	11.5 (3.4)	13.0 (3.0)	9.5 (2.4)	15.7 (2.9)
Monounsaturated fat intake*	13.6 (2.8)	19.8 (3.9)	13.2 (3.0)	17.4 (3.9)	14.4 (4.1)	16.9 (3.1)
Polyunsaturated fat intake*	5.4 (1.7)	5.4 (1.6)	3.5 (0.6)	7.6 (1.3)	5.1 (1.7)	5.3 (1.5)
*Trans* unsaturated fat intake*	0.4 (0.2)	0.3 (0.2)	0.3 (0.2)	0.4 (0.2)	0.2 (0.1)	0.6 (0.2)

OO: Olive oil; PUFA: Polyunsaturated fatty acids; TFA: Trans unsaturated fatty acids.

*Percentage of total energy intake.

Intakes of the different fat subtypes were positively correlated. Intake of SFA was correlated with MUFA, PUFA and TFA (Pearson correlation coefficients: 0.52, 0.15 and 0.73, respectively). Total fat intake contributed for 36.7% of total energy intake. MUFA was the major contributor to total energy intake (15.7%). The lowest contribution was TFA (only 0.4%).

Overall, 657 incident cases of depression were identified during the follow-up period with a total accrual of 68,694 person years. Table S1 shows the association between fat intake and depression risk. In multivariate-adjusted models, a significant inverse association was found for the fifth vs. the first quintile of PUFA intake with depression risk. Moreover, a significant inverse linear trend was observed (p = 0.031). In fully adjusted model, a borderline significant inverse dose-response relationship was observed for MUFA intake (p for trend = 0.053). Contrarily, TFA was associated with a higher risk. A substantial apparent relative increment (48%) in depression risk was found for the fifth vs. the first quintile of TFA, with a significant linear trend test (p = 0.003). After adjusting for adherence to the MDP, the association remained statistically significant (p for trend = 0.027).

Whereas butter consumption seemed to be associated with an increased risk of depression, OO consumption was inversely associated with depression risk (p for trend = 0.030). Even after adjusting for the adherence to the MDP, a borderline significant inverse trend remained for OO (p = 0.06) (Table 2).

**Table 2:** Association between culinary fats consumption (quintiles: Q1 to Q5) and depression*.

**Olive oil intake (g/d) (%)†**	2.8 (1.2)	7.3 (2.9)	11.0 (4.1)	20.6 (7.2)	28.0 (12.1)	
cases/person-years	143/13,933	126/13,788	137/13,619	118/13,867	133/13,486	
Crude rates/10^3^‡	10.3 (8.7–12.1)	9.1 (7.7–10.9)	10.1 (8.5–11.9)	8.5 (7.1–10.2)	9.9 (8.3–11.7)	
Multivariate-adjusted model (1)	1 (ref)	0.77 (0.60–1.00)	0.85 (0.65–1.10)	0.64 (0.49–0.84)	0.78 (0.60–1.01)	0.030
Additionally adjusted for MDP (2)	1 (ref)	0.78 (0.60–1.01)	0.87 (0.68–1.13)	0.66 (0.51–0.86)	0.80 (0.62–1.04)	0.060
**Butter intake (g/d) (%)†**	0 (0)	0.2 (0.1)	0.5 (0.1)	1.0 (0.4)	4.0 (1.3)	
cases/person-years	128/13,976	124/13,569	132/13,510	130/13,625	143/14,014	
Crude rates/10^3^‡	9.2 (7.7–10.9)	9.1 (7.7–10.9)	9.8 (8.2–11.6)	9.5 (8.0–11.3)	10.2 (8.7–12.0)	
Multivariate-adjusted model (1)	1 (ref)	1.07 (0.81–1.42)	1.23 (0.92–1.64)	1.16 (0.87–1.54)	1.30 (1.00–1.70)	0.076
Additionally adjusted for MDP (2)	1 (ref)	1.08 (0.81–1.42)	1.22 (0.92–1.63)	1.15 (0.86–1.53)	1.27 (0.97–1.67)	0.125

The SUN cohort 1999–2010.

Finally, no association was observed for n-3, n-6, n-3 from fish or the ratio between n-3 *vs* n-6 fatty acids intake. Neither was found for seed oils or margarine (data not shown).

Several sensitivity analyses were carried out to assess possible sources of bias in the estimation of the association between TFA intake and depression risk (Table 3). We repeated the analyses after excluding cases of depression reported in the first 2 years of follow up (n = 257) and the magnitude of the association for the fifth vs. first quintile of intake was even stronger. After the exclusion of cases reported after more than 8 years of follow-up (n = 54) or cases defined only by antidepressant use (n = 174), the association between TFA intake and depression risk remained of similar magnitude of effect (relative reduction in risk around 25–30%) though the conventional level of statistical significance was lost in some models.

**Table 3:** Sensitivity analysis for the association between the several dietary fats intake (quintiles) and depression.*

*Trans* unsaturated fat intake	Q1	Q2	Q3	Q4	Q5	P for trend
**Excluding early cases (n = 11,802; cases = 400)**						
Multivariate-adjusted (1)Additionally adjusted for MDP (2)	1 (ref)1 (ref)	1.37 (0.93–2.00)1.36 (0.92–1.99)	1.56 (1.07–2.26)1.54 (1.05–2.24)	1.67 (1.15–2.41)1.64 (1.12–2.39)	1.86 (1.30–2.66)1.81 (1.24–2.65)	0.0010.003
**Excluding late cases (n = 12,005; cases = 603)**						
Multivariate-adjusted (1)Additionally adjusted for MDP (2)	1 (ref)1 (ref)	1.06 (0.80–1.41)1.04 (0.78–1.38)	1.07 (0.80–1.42)1.03 (0.77–1.38)	1.21 (0.92–1.60)1.16 (0.87–1.55)	1.33 (1.01–1.74)1.25 (0.93–1.67)	0.020.09
**Excluding only antidepressant users (n = 11,885; cases = 483)**						
Multivariate-adjusted (1)Additionally adjusted for MDP (2)	1 (ref)1 (ref)	1.11 (0.80–1.53)1.09 (0.79–1.51)	1.14 (0.83–1.57)1.12 (0.81–1.55)	1.19 (0.87–1.63)1.16 (0.83–1.60)	1.33 (0.98–1.81)1.28 (0.92–1.78)	0.060.13

TFA: Trans unsaturated fatty acids; MDP: Mediterranean Dietary Pattern.

*Hazard ratios estimated with Cox Regression and 95% confidence interval (95% CI) If the confidence interval does not include 1.00, the results are statistically significant (two-tailed p<0.05).

(1): Model 1 additionally adjusted for smoking (non, former, current smoker and missing value), leisure time physical activity (in quintiles of MET score), total energy intake (Kcal/day), and BMI (Kg/m^2^).

(2): Model 2: Model 1 additionally adjusted for adherence to the Mediterranean Dietary Pattern (excluding SFA/MUFA ratio).

## Discussion

A direct potentially harmful association of TFA intake with the risk of depression was found in this Mediterranean cohort. The magnitude of this association was robust and persisted after several degrees of control for confounding and several sensitivity analyses. We also showed an inverse dose-response relationship for total PUFA and MUFA intake. An inverse association between olive oil consumption and depression was also found, although this relationship was attenuated after adjustment for the adherence to a Mediterranean Dietary Pattern.

To our knowledge, this is the first cohort study that has analyzed such a broad spectrum of fat subtypes in relation to depression risk. In consistency with our results, two previous smaller cohort studies reported inverse associations between OO or oleic acid intake and depressive symptoms or depression risk [28], [29]. The positive association for PUFA intake reported in those cohorts was weak and might be related to methodological issues. The first cohort [28] did not use clinical depression as outcome, but only assessed changes in a 15-point geriatric depression scale. The other cohort [29] had a relatively small sample size, high attrition rates and used a dietary assessment method (a 24-hour dietary recall), which is not considered the most adequate tool to assess usual diets.

Low-grade inflammatory status is frequently present among depressive patients [40], [41]. Pro-inflammatory cytokines may interfere with neurotransmitter metabolism, decrease plasma tryptophan level, alter neurotransmitter messenger RNA, and inhibit Brain-Derived-Neurotrophic-Factor (BDNF) expression [42], [43]. BDNF is a peptide critical for axonal growth, neuronal survival, and synaptic plasticity and function. BDNF levels have been reported to be reduced in patients with depression, and antidepressants seem to up-regulate BDNF and other neurotrophic and growth factors [44]. Neuroprotection is thought to be partly mediated by endothelial-produced BDNF [45]. A profile of fat subtypes that can promote a better endothelial function is thought to protect against neuropsychological disorders, including depression.

Our findings suggest that TFA intake, a well known risk factor for CVD, might have also a detrimental effect on depression. Depression and CVD seem to share some common mechanisms leading to similar biological changes [46], [47]. The adverse effects of TFA on CVD are thought to be mediated by increases in plasma concentrations of LDL-cholesterol, reductions in HDL-cholesterol, pro-inflammatory changes, endothelial dysfunction, and possibly by insulin resistance and displacement of essential fatty acids from membranes [48], [49]. Thus, since depression is associated with modifications in proinflammatory cytokines and also with endothelial cell signaling cascades alteration [50] (endothelium is responsible for the synthesis and secretion of BDNF [45], [51]), some detrimental biological modifications caused by TFA with respect to CVD risk could also be responsible for a harmful effect of TFA on depression risk. To our knowledge, the association between TFA and depression risk had not been reported before. Our results support a relationship between fat subtypes and depression which may parallel the well known effects of the quality of lipid intake on CVD risk [23]. The present analysis was carried out among a sample in which TFA intake was fairly low and where the two main sources of TFA intake were natural foods (cheese and whole-fat milk accounting both for 60% of the variability in total TFA intake, R^2^ = 0.60). In spite of the low absolute average intake of TFA in this cohort (TFA contributed only for a very small percentage of total energy, approximately only 0.4%) a substantial relative increment (48%) in depression risk was found for the highest category of intake. So, the repercussion of these results might be really important in other settings such as the American population where TFA intake is by far higher (up to 2.5% of total energy intake) [52], [53] and in which the main sources of TFA are artificial foods [54].

On the other hand, OO contains some bioactive polyphenols with important anti-inflammatory properties [55]. The anti-inflammatory capacity of OO could improve the function of the endothelium [56]–[58]. Moreover, the antioxidant actions of extra virgin OO components such as tyrosol are capable to restore the intracellular antioxidant defences [59] decreased among depressive patients. In fact, oxidative stress is associated to this pathology [60]. Oleic acid represents approximately 72 percent of OO [55]. From oleic acid, the lipid oleamide can be biosynthesized. Oleamide has important actions related to mood disorders, such as the induction of sleep [61]. It also plays a role in the maintenance of the physicochemical properties of membranes via its ability to increase the delta-9 desaturase enzyme activity and thus improving the binding of serotonin to its receptors [26], [62].

Adherence to the MDP has been associated with lower depression risk in a shorter follow-up of this cohort including only 480 incident cases [18]. It could be thought that an elevated adherence to the MDP among high OO users may at least partially explain some of our results. Although some potential overlaps between these results and those published before by our cohort could have occurred, we do not consider this possibility as probable, because we did not use at all TFA or olive oil as part of the definition of MDP in our previous report. Moreover, only small changes in the results were observed when the analyses were adjusted for adherence to the MDP. The adjustment for the adherence to this dietary pattern strengthens our results as we were able to independently assess the contribution of particular lipids to depression risk.

Unlike OO, butter, seed oils and margarine consumption was very low in this population, resulting in low between-subject variability and this lower consumption might contribute to explain that no associations were found for them.

The lack of association between n-3, n-6, n-3 from fish and depression risk, or between the ratio n-3:n-6 and depression risk in our cohort has also been documented in recent literature downplaying the importance of these fat subtypes or their ratio on depression [63], [64].

The dietary data analyzed in our study were exclusively those assessed at baseline (one time point only). The nature of this information imposed some constraints on the level of analysis that we could accomplish. We acknowledge that we did not account for any variation in dietary intake during the follow-up period. Further studies are needed to complete this assessment using repeated measurements of diet. Other potential limitations of our study need to be addressed, such as the self-reporting of a clinical diagnosis or the use of medication to define an incident case of depression. However, since participants were highly educated and committed to collaborate, we assumed that the proportion of misreporting the diagnosis would be low. Our validation study found very high specificity (0.96) for the self-reported diagnosis of depression. Theoretically, with perfect specificity, even when non-differential misclassification of disease because of low sensitivity exists, this will not bias the relative risk estimate [65]. Although the validity and reliability of the food-frequency questionnaire have been extensively evaluated, some degree of misclassification may exist in the dietary assessment and over- or under-estimation of true intake could have biased the estimates. However, being the miss-classification most probably non-differential, the bias will be probably towards the null. More importantly, participants with sub-clinical depression at baseline might have changed their food habits because of their mood disorder. But we believe that it is not likely that reverse causality could have biased our results because the exclusion of cases diagnosed during the first two years even strengthened the magnitude of the observed association. On the other hand, diet was collected at baseline and some of the cases of depression were reported after 10 years of follow-up. For that reason, the induction period for diet effect might be shorter than the time of follow-up of these late cases. Nevertheless, the exclusion of cases diagnosed after 8 or 10 years did not substantially change the magnitude of the association.

Several strengths of our study also deserve to be mentioned, such as its large sample size, its prospective design, the high retention rate in a long-term follow-up, the multiple adjustments of our estimates for potential confounders, the existence of published validation studies of our methods, and the population of highly educated participants who are able to provide information of high quality.

In conclusion, our results showed a protection of cardioprotective fats (PUFA and MUFA) and a detrimental effect of TFA on depression risk. However, our findings need to be confirmed by further prospective studies and by trials.

## Supporting Information

Table S1 **MDP: Mediterranean Dietary Pattern.** * Hazard ratios estimated with Cox Regression and 95% confidence interval (95% CI) If the confidence interval does not include 1.00, the results are statistically significant (two-tailed p<0.05). † energy adjusted median intake and percentage of total energy intake. ‡ Crude rates and 95% confidence intervals. (1) Model 1: adjusted for: sex, age (years), smoking (non, former, current smoker and missing value), leisure time physical activity (in quintiles of MET score), total energy intake (Kcal/day), and BMI (Kg/m^2^). (2) Model 2: Model 1 additionally adjusted for adherence to the Mediterranean Dietary Pattern (excluding SFA/MUFA ratio).
(DOC)
