# Supplementary material for: Dietary Fat Intake and the Risk of Depression: The SUN Project
Source: PLoS One. 2011 Jan 26;6(1):e16268. doi: 10.1371/journal.pone.0016268 (PMC3027671; doi:10.1371/journal.pone.0016268)
Supplement: Table S1 — MDP: Mediterranean Dietary Pattern. * Hazard ratios estimated with Cox Regression and 95% confidence interval (95% CI) If the confidence interval does not include 1.00, the results are statistically significant (two-tailed p<0.05). † energy adjusted median intake and percentage of total energy intake. ‡ Crude rates and 95% confidence intervals. (1) Model 1: adjusted for: sex, age (years), smoking (non, former, current smoker and missing value), leisure time physical activity (in quintiles of MET score), total energy intake (Kcal/day), and BMI (Kg/m2). (2) Model 2: Model 1 additionally adjusted for adherence to the Mediterranean Dietary Pattern (excluding SFA/MUFA ratio). (DOC) [file pone.0016268.s001.doc]

**Table 2. Association between dietary fat intake** **(quintiles: Q1 to Q5) and depression*. The SUN cohort 1999-2010.**

| **Fat type** | **Q1** | **Q2** | **Q3** | **Q4** | **Q5** | **P for trend** |
| --- | --- | --- | --- | --- | --- | --- |
| **Total fat intake (g/d) (%)**† | 75.5 (28.5) | 88.5 (33.5) | 96.2 (36.6) | 104.0 (39.7) | 117.1 (44.6) |  |
| cases/person –years | 134/13,020 | 125/13,317 | 130/13,902 | 136/14,110 | 132/14,344 |  |
| Crude rates/10³‡ | 10.3 (8.7-12.2) | 9.4 (7.9-11.2) | 9.4 (7.9-11.1) | 9.6 (8.1-11.4) | 9.2 (7.8-10.9) |  |
| Multivariate-adjusted model (1) | 1 (ref) | 0.88 (0.68-1.14) | 0.92 (0.71-1.20) | 0.91 (0.71-1.18) | 0.90 (0.70-1.17) | 0.53 |
| Additionally adjusted for MDP (2) | 1 (ref) | 0.85 (0.65-1.10) | 0.86 (0.66-1.12) | 0.83 (0.63-1.08) | 0.79 (0.60-1.04) | 0.11 |
| **Saturated fat intake (g/d) (%)**† | 23.0 (8.7) | 28.9 (10.9) | 32.7 (12.4) | 36.5 (13.9) | 42.8 (16.4) |  |
| cases/person-years | 115/12,801 | 138/13,496 | 115/13,885 | 144/14,118 | 145/14,394 |  |
| Crude rates/10³‡ | 9.0 (7.5-10.8) | 10.2 (8.7-12.1) | 8.3 (6.9-9.9) | 10.2 (8.7-12.0) | 10.1 (8.6-11.9) |  |
| Multivariate-adjusted model (1) | 1 (ref) | 1.20 (0.92-1.56) | 0.95 (0.71-1.25) | 1.23 (0.95-1.61) | 1.23 (0.95-1.61) | 0.12 |
| Additionally adjusted for MDP (2) | 1 (ref) | 1.16 (0.89-1.52) | 0.90 (0.67-1.19) | 1.14 (0.86-1.51) | 1.11 (0.83-1.48) | 0.56 |
| **Monounsaturated fat intake (g/d) (%)**† | 30.3 (11.5) | 36.5 (13.8) | 40.4 (15.4) | 44.9 (17.2) | 52.9 (20.2) |  |
| cases/person-years | 138/13,062 | 125/13,479 | 137/13,835 | 121/14,114 | 136/14,203 |  |
| Crude rates/10³‡ | 10.6 (8.9-12.5) | 9.3 (7.8-11.1) | 9.9 (8.4-11.7) | 8.6 (7.2-10.2) | 9.6 (8.1-11.3) |  |
| Multivariate-adjusted model (1) | 1 (ref) | 0.87 (0.68-1.13) | 0.89 (0.69-1.14) | 0.74 (0.57-0.96) | 0.87 (0.68-1.11) | 0.17 |
| Additionally adjusted for MDP (2) | 1 (ref) | 0.85 (0.65-1.09) | 0.84 (0.65-1.09) | 0.69 (0.53-0.90) | 0.80 (0.62-1.03) | 0.05 |

**Table 2 (cont.). Association between dietary fat intake** **(quintiles: Q1 to Q5) and depression*. The SUN cohort 1999-2010.**

| **Fat type** | **Q1** | **Q2** | **Q3** | **Q4** | **Q5** | **P for trend** |
| --- | --- | --- | --- | --- | --- | --- |
| **Polyunsaturated fat intake (g/d) (%)**† | 9.3 (3.6) | 11.7 (4.3) | 13.4 (5.0) | 15.3 (5.8) | 19.0 (7.2) |  |
| cases/person-years | 141/13,277 | 135/13,495 | 144/13,534 | 125/14,022 | 112/14,366 |  |
| Crude rates/10³‡ | 10.6 (9.0-12.5) | 10.0 (8.5-11.8) | 10.6 (9.0-12.5) | 8.9 (7.5-10.6) | 7.8 (6.5-9.4) |  |
| Multivariate-adjusted model (1) | 1 (ref) | 0.96 (0.74-1.24) | 1.02 (0.79-1.32) | 0.90 (0.70-1.17) | 0.80 (0.62-1.04) | 0.07 |
| Additionally adjusted for MDP (2) | 1 (ref) | 0.95 (0.73-1.22) | 1.0 (0.78-1.29) | 0.88 (0.67-1.14) | 0.76 (0.59-0.99) | 0.03 |
| ***Trans* unsaturated fat intake (g/d) (%)**† | 0.4 (0.1) | 0.7 (0.3) | 0.9 (0.3) | 1.2 (0.4) | 1.6 (0.6) |  |
| cases/ person-years | 111/12,748 | 117/13,411 | 129/13,648 | 141/14,282 | 159/14,605 |  |
| Crude rates/10³‡ | 8.7 (7.2-10.5) | 8.7 (7.3-10.5) | 9.5 (8.0-11.2) | 9.9 (8.4-11.6) | 10.9 (9.3-12.7) |  |
| Multivariate-adjusted model (1) | 1 (ref) | 1.08 (0.82-1.43) | 1.17 (0.88-1.53) | 1.28 (0.97-1.68) | 1.42 (1.09-1.84) | 0.003 |
| Additionally adjusted for MDP (2) | 1 (ref) | 1.06 (0.80-1.40) | 1.13 (0.85-1.49) | 1.22 (0.92-1.62) | 1.33 (1.00-1.76) | 0.03 |
